# Psychometric Evaluation of a Thai Version of the Cardiac Distress Inventory

**DOI:** 10.1111/nicc.70605

**Published:** 2026-08-06

**Authors:** Nittaya Srisuk, Naruebeth Koson, Arunsri Rattanaprom, Nattiya Peansungnern, Michael R. Le Grande, Alun C. Jackson, David R. Thompson, Chantal F. Ski

**Affiliations:** ^1^ Faculty of Nursing Rajamangala University of Technology Thanyaburi Pathum Thani Thailand; ^2^ Ministry of Public Health Boromarajonani College of Nursing, Faculty of Nursing, Praboromarajchanok Institute Nakhon Si Thammarat Thailand; ^3^ Cardiac Care Unit Surat Thani Hospital Surat Thani Thailand; ^4^ Ministry of Public Health Boromarajonani College of Nursing, Faculty of Nursing, Praboromarajchanok Institute Nakhon Ratchasima Thailand; ^5^ Australian Centre for Heart Health Melbourne Australia; ^6^ School of Psychological Sciences The University of Melbourne Melbourne Australia; ^7^ School of Nursing and Midwifery Queen's University Belfast Belfast UK

**Keywords:** cardiac distress inventory, cultural adaptation, reliability, Thai, validity

## Abstract

Cardiac distress is common, yet minimal support is available for nurse assessment of patients. We translated the Cardiac Distress Inventory (CDI) into Thai (CDI‐Thai) and evaluated its psychometric properties. This was a cross‐sectional study of 250 adults who experienced an acute cardiac event in the past year in southern Thailand. The CDI was translated into Thai using forward–backward translation and expert review. The CDI‐Thai achieved translation equivalence to the original version. An eight‐factor structure was confirmed by factor analysis, with excellent fit. The CDI‐Thai was shown to be culturally relevant and psychometrically robust as a measure of cardiac distress.

## Impact Statements

• What is known about the topic

○ Psychological distress is common in patients with cardiac disease, yet minimal support is available for nurse assessment.

• What this paper adds

○ The CDI‐Thai emerged as a culturally attuned, psychometrically robust instrument for assessing distress in Thai cardiac patients.
○ The CDI‐Thai can be used to support nurses in their assessment and management of psychological distress in cardiac patients.

## 1 Background

In Thailand, cardiovascular disease remains in the top five leading causes of death and is strongly associated with premature mortality and disability‐adjusted life years [1]. Anxiety, depression and stress are common conditions occurring after an acute cardiac event [2]. A recent systematic review and meta‐analysis estimated the global prevalence of depression at 31.3%, anxiety at 32.9% and stress levels at 57.7% in cardiac patients [3]. Studies in Thailand have reported one in four patients with acute myocardial infarction experiencing post‐traumatic stress disorder and strong associations with symptoms of anxiety, depression and helplessness [4, 5]. Acknowledging the multidimensional psychological impact following a cardiac event, Jackson and colleagues proposed the concept of ‘cardiac distress’ and defined it as a persistent negative emotional state that challenges the capacity of a patient to cope with their heart condition, associated treatments and resultant changes to daily living [6].

Numerous international guidelines and advisories have recommended screening of psychological stressors for those with cardiac conditions for example American Heart Association [7], British Association for Cardiovascular Prevention and Rehabilitation [8], European Society of Cardiology [9]. However, psychological distress in cardiac patients is most commonly assessed using a combination of measurement tools including: depression (e.g., Patient Health Questionnaire 9 [10]; Cardiac Depression Scale [11]), anxiety (e.g., Generalized Anxiety Disorder 7 [12]; Cardiac Anxiety Questionnaire [13]) and stress (e.g., Perceived Stress Scale) [14]. In contrast, the Cardiac Distress Inventory (CDI) was developed to provide a comprehensive assessment of ‘cardiac distress’ across multiple domains in a single measure [15].

The CDI measures responses to physical, affective, cognitive, behavioural and social symptoms and experiences related to a patient's cardiac event and recovery [15]. Development and validation of the CDI in an Australian population (*n* = 405) confirmed a 55‐item measure with eight subscales that demonstrated good psychometric properties in terms of validity and reliability [16]. To date, there is no culturally adapted, psychometrically validated measure of cardiac distress for Thai patients. The purpose of this study was to translate the CDI into Thai and evaluate its psychometric properties in a sample of Thai patients with heart disease.

## 2 Methods

This cross‐sectional study recruited patients between December 2022 and February 2023. The study followed the International Society for Pharmacoeconomics and Outcomes Research (ISPOR) recommendations for translation and cross‐cultural adaptation of patient‐reported outcome measures [17].

A convenience sample of community‐dwelling adult outpatients was recruited through a cardiac rehabilitation clinic at a tertiary hospital in southern Thailand. Inclusion criteria were: (i) ≥ 18 years; and (ii) diagnosis of cardiac disease within the preceding 12 months. Exclusion criteria were: (i) documented cognitive dysfunction, severe mental illness or frailty based on medical history and clinical records; and (ii) insufficient Thai language proficiency. Eligible participants were identified through review of electronic health records and approached by the research team in the outpatient clinic. Participants who met the inclusion criteria were provided with a written description of the study. Those who consented completed a series of questionnaires taking approximately 30 min to complete.

Three measures were included. A brief sociodemographic and clinical characteristic survey was used to collect participant information. The Emotion Thermometers Thai version (ET‐Thai) comprises four thermometers: distress (DT), anxiety (AnxT), depression (DepT) and anger (AngT) and has confirmed concurrent and criterion validity [18]. Also, the Cardiac Distress Inventory (CDI) comprised 55 items distributed across eight subscales: ‘Fear and uncertainty’, ‘Disconnection and hopelessness’, ‘Changes to roles and relationships’, ‘Overwhelm and depletion’, ‘Cognitive challenges’, ‘Physical challenges’, ‘Health system challenges’ and ‘Death concerns’ [16]. Items are scored on a scale of 1 to 4. Total score ranges from 0 to 165, with a cut‐off score of ≥ 25 indicating moderate cardiac distress and ≥ 37 indicating severe cardiac distress [16].

The CDI was translated into Thai and cross‐culturally adapted using forward and backward translation and standard guidelines on scale adaptation [19]. This included avoidance of literal or word‐for‐word translation if there was potential for compromise of the clarity or cultural relevance. Rather, language adaptations were made to achieve conceptual, semantic and content equivalence [19]. Two independent bilingual translators completed forward translation, followed by synthesis into a reconciled Thai version. Two additional bilingual translators blinded to the original instrument subsequently performed backward translation. An expert panel, including a doctoral‐prepared nurse, a non‐medical professional and specialists in nursing management, maternity care and medical English, evaluated semantic equivalence, clarity and cultural appropriateness using the content validity index (CVI) [20]. To minimise translation bias, translators and the expert panel responsible for content validity evaluation were different individuals. Cognitive interviews, to explain why participants chose a specific answer to reveal any confusion or misinterpretation of the questions, were conducted with 10 Thai cardiac patients (diagnosed with cardiac disease in past 12 months) to assess comprehensibility prior to psychometric testing, resulting in minor linguistic refinements while preserving conceptual equivalence with the original instrument.

A content validity index (CVI) was used to identify potential cultural differences from forward and backward translation [20]. Given the CDI's consistent eight‐factor structure, confirmatory factor analysis (CFA) was used to evaluate construct validity. Descriptive analyses, content validity indices and concurrent validity were analysed using SPSS version 28. Robust maximum likelihood CFA and associated reliability estimates were performed in LISREL 8.72 and cross‐validated using Stata/BE 17. Adequate model fit was defined as χ^2^/df ≤ 3, comparative fit index (CFI) ≥ 0.90, incremental fit index (IFI) ≥ 0.90, normed fit index (NFI) ≥ 0.90, adjusted goodness‐of‐fit index (AGFI) ≥ 0.90, root mean square error of approximation (RMSEA) ≤ 0.08 with a 90% confidence interval ≤ 0.10 and standardised root mean square residual (SRMR) ≤ 0.08 [21]. Convergent validity was assessed using factor loadings (≥ 0.40 sufficient; ≥ 0.70 highly commendable) [22] average variance extracted (AVE) ≥ 0.50 and composite reliability (CR) ≥ 0.70 [23]. Discriminant validity was established when the square root of each factor's AVE exceeded inter‐factor correlations and further using heterotrait–monotrait ratio of correlations (HTMT; < 0.85) [24]. Concurrent validity was assessed using Pearson's correlation with the ET‐Thai scales. Internal consistency was evaluated using Cronbach's alpha. Sample size for CFA ranges from 2 to 20 subjects per item [22]. The CDI comprises 55 items; thus, a sample size of 250 was deemed adequate for CFA.

Ethical approval was obtained from the Institutional Review Board of Suratthank Hospital (REC65‐0074; 05.11.2022). This study adhered to the principles outlined in the Declaration of Helsinki.

## 3 Results

The sample comprised a total of 250 participants (179 male) with an average age of 59. Most were married (79.6%) with New York Heart Classification of I or II (95.6%). Translational validity (CVI) for the 8 factors of the CDI‐Thai ranged between, *k* = 0.96–0.99 with overall scale validity at *k* = 0.98. Confirmatory factor analysis supported the eight‐subscale structure of CDI‐Thai, with each model exhibiting excellent fit to the data (*χ* ^2^/df = 1.04–2.47; CFI = 0.93–0.99; IFI = 0.93–0.99; NFI = 0.91–0.98; AGFI = 0.90–0.96; RMSEA = 0.024–0.069; SRMR = 0.021–0.056). Fit indices for each of the eight single‐factor models are shown in Table 1.

**TABLE 1:** Fit index values for the CDI‐Thai.

CDI‐Thai domains	*χ* ^2^ Goodness of fit				CFI	GFI	AGFI	RMSEA	RMR
	*χ* ^2^	df	*χ* ^2^/df	*p*					
*Single‐factor CFAs*									
FACTOR 1—Fear and uncertainty	3.76	13	1.05	0.390	1.00	0.99	0.96	0.015	0.014
FACTOR 2—Disconnection and hopelessness	14.57	14	1.04	0.408	1.00	0.99	0.96	0.013	0.007
FACTOR 3—Changes to roles and relationships	38.36	30	1.28	0.141	0.99	0.97	0.94	0.033	0.016
FACTOR 4—Overwhelm and depletion	9.81	12	0.82	0.632	1.00	0.99	0.97	0.000	0.015
FACTOR 5—Cognitive challenges	0.15	1	0.15	0.699	1.00	1.00	1.00	0.000	0.002
FACTOR 6—Physical challenges	13.32	10	1.33	0.206	1.00	0.99	0.95	0.037	0.013
FACTOR 7—Health‐system challenges	6.12	4	1.53	0.190	1.00	0.99	0.96	0.046	0.012
FACTOR 8—Death concern	0.03	1	0.03	0.871	1.00	1.00	1.00	0.000	0.001
*Full eight‐factor model*									
CDI‐Thai (Overall)	919.31	872	1.05	0.129	0.99	0.95	0.91	0.015	0.040

*Note:* Value criteria: χ^2^/df < 2.00; *p* > 0.05; CFI, GFI, AGFI ≥ 0.90; RMSEA ≤ 00.05; RMR ≤ 0.05.

Abbreviations: AGFI, adjusted goodness‐of‐fit index; CDI‐Thai, cardiac distress inventory Thai version; CFA, confirmatory factor analysis; CFI, comparative fit index; IFI, incremental fit index; NFI, normed fit index; RMR, root mean square residual; RMSEA, root mean square error of approximation.

Standardised factor loadings ranged from 0.18 to 0.94. Of the 55 items, 44 (80%) loaded ≥ 0.50 and seven loaded between 0.40 and 0.49. Three items failed to reach the minimum threshold: two from the ‘Changes to roles and relationships’ subscale, CDI44 (0.29) and CDI55 (0.18) and one from the ‘Cognitive challenges’ subscale CDI51 (0.20). Composite reliability and McDonald's omega ranged from 0.70 to 0.88 across subscales, and Cronbach's alpha varied between 0.75 and 0.87 confirming acceptable to good internal consistency. Average variance extracted (AVE) exceeded the recommended 0.50 cut‐off for Disconnection and hopelessness (0.52) and Health‐system challenges (0.61); the remaining subscales yielded values between 0.28–0.46, reflecting heterogeneous item clustering. All subscales met the dual criteria of CR ≥ 0.70 and AVE > 0.40 (Table 2) considered sufficient convergent evidence for newly translated instruments. All pairwise HTMT values ranged between 0.18–0.67, and bias‐corrected 95% confidence intervals were < 0.90 indicating satisfactory discriminant validity across all subscales, for example the highest HTMT coefficient emerged between Overwhelm and depletion and Cognitive challenges (0.66); below the 0.85 threshold reflecting clear conceptual separation.

**TABLE 2:** Factor‐level summary of loadings, AVE, CR and internal consistency reliability for the CDI‐Thai (*N* = 250).

Subscale	Items	Standardised loading range	Mean standardised loading	AVE	CR	Cronbach's alpha	McDonald's omega
Health‐system challenges	5	0.21–0.92	0.73	0.61	0.87	0.85	0.87
Disconnection and hopelessness	8	0.06–0.85	0.68	0.52	0.88	0.87	0.88
Death concern	4	0.56–0.78	0.67	0.46	0.77	0.77	0.77
Cognitive challenges	4	0.20–0.94	0.60	0.43	0.72	0.73	0.72
Physical challenges	8	0.41–0.73	0.58	0.35*	0.81	0.82	0.81
Fear and uncertainty	8	0.33–0.69	0.56	0.33*	0.79	0.78	0.79
Changes to roles & relationships	11	0.18–0.77	0.54	0.32*	0.82	0.85	0.82
Overwhelm and depletion	7	0.21–0.72	0.49	0.28*	0.70	0.75	0.70

Abbreviations: AVE, average variance extracted; CR, composite reliability.

* AVE < 0.40: b)ut CR ≥ 0.70; acceptable for newly translated instruments.

Corrected item‐total correlations were satisfactory for 48 items (≥ 0.40) of 55 items. Three items exhibited low correlations; CDI5 (0.13), CDI22 (0.21) and CDI51 (0.35). These items were retained as alpha‐if‐deleted analysis showed removing CDI5 increased Cronbach alpha for ‘Disconnection and hopelessness’ from 0.87 to 0.89, while deleting CDI43 or CDI55 increased alpha by 0.02 to 0.03. Lastly, Significant correlations were found for all four ET's, for example Anxiety (*r* = 0.612, *p* < 0.001) and Distress (*r* = 0.598, *p* < 0.001) demonstrating concurrent validity.

## 4 Discussion

Our findings demonstrate successful translation of the CDI into Thai through expert‐consensus of content validity and translational equivalence. Sound psychometric properties of the CDI‐Thai were also confirmed in a cardiac population in Southern Thailand. With minimal resources available for psychological assessment of patients by nurses [25], the CDI‐Thai fills an established knowledge‐to‐practice gap that will aid nurses in their assessment and provision of psychological support for patients with cardiac disease. Timely identification of psychological distress is crucial for enhancing patient outcomes and facilitating early intervention.

The CFA yielded an excellent fit for its multidimensional structure and scale reliability was evidenced by internal consistency, composite reliability and McDonald's omega. Our assessment supports the CDI‐Thai's multidimensional structure for assessing cardiac distress, as per the original CDI subscales: fear and uncertainty; disconnection and hopelessness; changes to roles and relationships; overwhelm and depletion; cognitive challenges; physical challenges; health‐system challenges; and death concerns. Overall, the CDI‐Thai has shown to be a valid and reliable measure of cardiac distress among Thai patients' post‐acute cardiac event, making it a useful resource for critical care nurses. These findings are consistent with the original validation of the CDI, which conceptualised cardiac distress as a multidimensional psychosocial response encompassing emotional, cognitive, behavioural, social and health‐system‐related challenges associated with cardiac illness and recovery [16].

Analysis of the CDI‐Thai's scale quality indicated acceptable to good convergent validity and reliability. Composite reliability and McDonald's omega ranged from 0.70 to 0.88, confirming strong internal consistency. The ‘Disconnection and hopelessness’ and ‘Health system challenges’ subscales achieved AVE values above 0.50. Although the remaining subscales exhibited AVE values between 0.28 and 0.46 which is below the conventional 0.50 threshold, they nonetheless met the dual criteria of CR ≥ 0.70 and AVE > 0.40 for newly translated instruments [23], in support of acceptable convergent validity. In terms of discriminant validity, HTMT ratios between subscales ranged from 0.18 to 0.67, all below the 0.85 cutoff [24], confirming each subscale measures a distinct construct. The highest HTMT observed between ‘Overwhelm and depletion’ and ‘Cognitive challenges’ (0.66) remained below the conservative 0.85 threshold, consistent with findings that patients experiencing overwhelm often also report increased cognitive load [24].

This study has some limitations that should be acknowledged. Data collection took place on a single occasion, and no test–retest evaluation was conducted, thereby precluding assessment of the instrument's stability. The use of a convenience sample introduces the possibility of selection bias, which restricts the generalisability of our findings. Consistent with recommendations for cross‐cultural adaptation of patient‐reported outcome measures, the CDI‐Thai incorporated standard Thai language [17]. However, participants in this study were recruited from southern Thailand, where regional dialects and sociocultural communication patterns may differ with potential for influencing interpretation. Of note, cognitive interviews and pilot testing provided good indication of adequate understanding of questionnaire items. Educational attainment may also have influenced questionnaire comprehension, as a substantial proportion of participants reported up to primary‐level education. Additionally, although the average questionnaire completion time was approximately 30 min, the maximum recorded time was 50 min. While the multidimensional structure of the CDI provides comprehensive assessment of cardiac distress, the length of the CDI‐Thai (55 items) may increase respondent burden, particularly among older adults. Development of a shorter screening version of the CDI‐Thai may therefore improve feasibility and acceptability in routine clinical practice and when caring for patients in acute care environments. Lastly, measurement invariance across subgroups such as age and sex was not evaluated, which could restrict interpretations of the instrument's equivalency.

## 5 Conclusion

The CDI‐Thai emerged as a culturally attuned, psychometrically robust instrument for assessing the complex, multidimensional distress experienced by Thai cardiac patients. Its validated eight‐subscale structure and strong reliability and validity indices support its use in both research and clinical practice. As cardiac patients commonly experience significant psychological distress, especially in the acute setting, timely assessment using the CDI‐Thai can support nurses in their assessment and management of these patients.

## Author Contributions

**Nittaya Srisuk:** conceptualisation, writing – original draft, investigation, data curation, project administration. **Naruebeth Koson:** writing – original draft, Project administration, Investigation, data curation. **Arunsri Rattanaprom:** writing – review and editing, investigation, project administration. **Nattiya Peansungnern:** writing – original draft, investigation, project administration, data curation. **Michael R. Le Grande:** methodology, data curation, formal analysis, writing – review and editing. **Alun C. Jackson:** conceptualisation, writing – review and editing. **David R. Thompson:** conceptualisation, supervision, methodology, writing – review and editing. **Chantal F. Ski:** conceptualisation, supervision, methodology, writing – review and editing.

## Funding

This research did not receive any specific grant from funding agencies in the public, commercial or not‐for‐profit sectors.

## Ethics Statement

Ethical clearance was granted by the Institutional Review Board of Suratthani Hospital (REC 65‐0074), 5 November 2022.

## Consent

All eligible participants were informed orally by the lead researchers (N.S.; N.K.) about the aims, content and procedure of the study. After providing the opportunity to ask questions, written consent was obtained from the participants.

## Conflicts of Interest

The authors declare no conflicts of interest.

## Data Availability

The data that support the findings of this study are available from the corresponding author upon reasonable request.
